# Efficient Approach for Direct Robust Surface Grafting of Polyethyleneimine onto a Polyester Surface during Moulding

**DOI:** 10.3390/polym16050644

**Published:** 2024-02-27

**Authors:** Philipp Zimmermann, Silven Frohs, Martin Wiesing, Kamal Meena, Jürgen Nagel

**Affiliations:** 1 Leibniz-Institut für Polymerforschung Dresden e.V., 01069 Dresden, Germany; zimmermann-philipp@ipfdd.de (P.Z.); meena@ipfdd.de (K.M.); 2 Fraunhofer-Institut für Fertigungstechnik und Angewandte Materialforschung, 28359 Bremen, Germany; martin.wiesing@ifam.fraunhofer.de

**Keywords:** surface modification, polymer chemistry, grafting-to, chemical stability, polyester, 3D printing

## Abstract

This paper uses a very effective way for surface modification of thermoplastic polymers during moulding. It is based on a grafting reaction between a thin layer of a functional polymer, deposited on a substrate in advance, and a polymer melt. In this paper, a glycol-modified polyethylene terephthalate (PETG) that was brought in contact with a polyethyleneimine layer during fused filament fabrication is investigated. The focus of this paper is the investigation of the reaction product. Grafting was realised by the formation of stable amide bonds by amidation of ester groups in the main chain of a PETG. XPS investigations revealed that the conversion of amino groups was very high, the distribution was even, and the quantity of amino groups per polyester surface area was still very high. The surface properties of the produced polyester part were mainly characterised by polyethyleneimine. The grafting was able to resist several cycles of extraction in alkaline solutions. The stability was only limited by saponification of the polyester. The degree of surface modification was dependent on the molar mass of polyethyleneimine. This could be rationalised, because grafting only occurred with the one polyethyleneimine molecule that is in close vicinity to the polyester surface when both components come in contact. Fused deposition modelling was chosen as the model process with control over each processing step. However, any other moulding process may be applied, particularly injection moulding for mass production.

## 1. Introduction

Polymer surfaces often need a chemical surface modification. Appropriate methods are widely described in the literature [1,2,3,4,5,6]. This is carried out in industry as well as in research facilities by several methods, e.g., those using any kind of plasma [7,8,9,10,11,12,13,14,15] and plasma deposition [16,17], or any layer deposition, like deposition of polyelectrolyte multilayers [18,19,20,21]. However, the most robust modifications are based on any type of grafting procedure, where a functional polymer is attached to the polymer substrate surface by chemical bonding. There are two ways of grafting on polymer surface: the grafting-from [22,23,24,25,26,27,28] and the grafting-to procedures [29,30,31]. The first step of the grafting-from procedure is the chemical bonding of a precursor molecule on the polymer surface. This is typically a polymerization initiator. In a second step, a polymerization is started from this precursor, resulting in formation of a polymer layer where every molecule is chemically bound to the polymer surface by exactly one bond. In contrast, the grafting-to approach starts from a functional polymer with reactive groups that can undergo a chemical coupling reaction with the polymer substrate. This coupling can be achieved by radical reactions or by specific coupling reactions with end groups of the polymer chain or groups within the polymer chain. Activation of these reactions can be realised by u. v. radiation [32,33,34,35,36], infrared laser radiation [37], or other [38].

However, all the mentioned approaches need a separate processing step for the grafting reaction. We have developed a method that carries out grafting during moulding of a thermoplastic part [39,40,41]. The method of process-integrated surface modification starts with the deposition of a thin layer of a functional polymer on a substrate. This substrate can be a steel plate, a silicon wafer for fundamental research, or the mould of an injection moulding machine in case of mass production. Spray coating, dip coating, spin coating, and inkjet printing were used for the deposition of thin films of functional polymers. The high temperature necessary for initiating the grafting reaction is only available for a short time because the melt cools down very fast on contact with the colder substrate. The reaction time is typically limited to approximately a micro second [42]. Consequently, only those polymer segments can react that are in close contact, as was found out by molecular simulations using the bond fluctuation model [43,44]. Nevertheless, grafting was achieved experimentally within the short time at high temperatures available during injection moulding [42].

In this contribution, 3D printing by Fused Filament Fabrication (FFF) was used for preparation of parts. During the process, the hot melt is exposed to the functional polymer at high temperature. The material system was used recently for micro structured functionalisation of a polyester surface, where first proofs of a reaction were established [45]. The system was chosen as a model because the amidation reaction between a polyester and a polyamine has been well investigated [46]. PETG is a material frequently used for FFF. The foci of this paper are (i) the investigation and quantification of the grafting reaction under the special processing conditions and (ii) the stability of the grafted functional polymer on the PETG surface. Hyper-branched Polyethyleneimine (PEI) was used as the functional polymer because it bears a high number of amino groups, particularly primary amino groups, which are more reactive that other amino groups. The idea was to use this highly functional PETG surface as a precursor, because amino groups are accessible for many reactive coupling reactions under mild conditions, e.g., for chemical bonding or metal plating. A requirement is that the amino groups withstand the thermal and oxidative processes. PEI is available in a broad range of molar masses. We have chosen the lowest molar mass as 2 kg mol^−1^ because PEI with a lower molar mass could evaporate during printing. The highest molar mass was 750 kg mol^−1^. This material is easily available and affordable for later technical use. The conversion of amino groups to amide groups was investigated for the first time by extensive XPS experiments. The functionalisation effect was evaluated by the adsorption behaviour of the anionic dye pyranine, which was used as a model for anionic compounds. Technical interesting anionic molecules could be proteins, inorganic and organic acids, or surfactants. Moreover, the amino-modified surface underwent typical reactions of amino groups, i.e., the amino groups were still active. The adsorption and complex formation with Pd ions is shown here as an example.

## 2. Experimental

### 2.1. Materials

Moulding experiments were carried out using a white PETG filament (Extrudr F3D GmbH, Vorarlberg, Austria) with a diameter of 1.75 mm during FFF printing. Microscopy glass slides with the dimensions of 1 in. × 3 in. and a thickness of 1 mm (Menzel GmbH, Berlin, Germany) served as substrates for printing. Hyper-branched PEI of different molar mass (SigmaAldrich SE, Steinheim, Germany, molar masses given from the supplier) were used as follows: PEI-750k (M_W_ = 750 kg mol^−1^, M_N_ = 60 kg mol^−1^, 50 percent by mass), PEI-25k (M_W_ = 25 kg mol^−1^, M_N_ = 10 kg mol^−1^), PEI-2k (M_W_ = 2 kg mol^−1^, 1.8 M_N_ = kg mol^−1^, solution 50 percent by mass). Pyranine dye as well as other laboratory chemicals (NaOH, HCl solution, ethanol, and borax) were purchased from SigmaAldrich SE (Steinheim, Germany).

### 2.2. Preparation of Solutions and Substrates

A pyranine solution (10^−3^ M) was prepared in borate buffer with a pH value of 9. A 1 M NaOH solution was prepared by dissolving the appropriate amount of NaOH in water. Concentrations of 10^−1^, 10^−2^, 10^-3^, 10^−4^, 10^−5^, and 10^−6^ M were prepared by stepwise dilution. The PEI solutions with different molar masses were prepared by mixing the appropriate amount of PEI in a mixture of water and ethanol (40 per cent by volume). The resulting concentrations were 50 g L^−1^ (PEI-750k) and 100 g L^−1^ of PEI (PEI-25k), respectively. The solution of PEI-2k was used without further dilution (500 g L^−1^). Thin films of the different PEI were spin coated from these solutions on the substrates using a Spin 150 Coater (SPS-Europe GmbH, Ingolstadt, Germany).

### 2.3. Moulding

Moulding was performed using a Raise 3D Pro 2 3D printer (Raise 3D Technologies, Irvine, CA, USA). The print bed was equipped with a recess where the glass slides fit in. A coated glass slide (for reference: a clean glass slide) was mounted in this recess before printing PETG parts on top with the dimensions 20 mm × 40 mm. Three layers were printed, resulting in a thickness of 0.4 mm. The extruder temperature was set to 280 °C and the print bed temperature to 100 °C. More details of the printing process were published recently [45].

### 2.4. Pyranine Staining and Extraction

All PETG-PEI samples were rinsed in water for 1 h under constant shaking using a lab shaker, changing the water every 20 min. The samples were put in a test tube filled with the pyranine solution and shaken for 10 min. Finally, the samples were rinsed with water to remove any excess pyranine. To measure the adsorbed amount of pyranine, the sample was extracted in alkaline solution. The extractions were performed with varying NaOH concentrations at room temperature by placing the samples in a test tube, filled with the alkaline solution for 1 h. The samples were then rinsed with water, which was added to the extract. The pH value of the extract was adjusted to 9 by adding HCl solution. Finally, the extract was poured into a volumetric flask and filled up with borate buffer. Optical spectra of these solutions were recorded with a Cary 60 Spectrometer (Agilent Technologies, Santa Clara, CA, USA) from 300 to 600 nm with water as reference. The maximum absorption of pyranine was at 455 nm. For quantitative analyses, the extinctions at this wavelength were evaluated. The amount of pyranine per area N′ extracted from a sample was calculated with Equation (1)

*N′ = v E/(ε d A)*, (1)

where *v* is the volume, *E* the measured extinction of the extract solution, *d* the thickness of the cuvette, and *A* the area of one surface of the printed part. *ε* is the extinction coefficient of pyranine, which was measured in advance over a broad concentration range as described in Supplementary S1.

### 2.5. Characterisation

Optical spectra of the solutions in cuvettes with a light path of 1 cm were performed with a Cary 60 spectrometer (Agilent, Santa Clara, CA, USA).

EDX mappings were recorded with a Phenom XL Workstation (Thermo Scientific, Waltham, MA, USA) equipped with a thermoelectrically cooled Silicon Drift Detector SDD with 25 mm^2^ detector area. Measurements were carried out using the beam intensity mapping, an accelerating voltage of 15 kV, and low vacuum mode (60 Pa). Mappings were recorded with a resolution of 480 pixels with 10 ms per pixel.

XPS has been measured using an Escalab 250Xi (Thermo Fisher, Waltham, MA, USA) equipped with a monochromatic Alkα source (1486.7 eV, 650 µm analysis spot). Neutralisation of the surface was carried out using a combination of low-energy electrons and Ar^+^ ions. The spectra were acquired in electrostatic mode at 150 eV constant analyser pass energy (CEA) for survey, and at 20 eV CAE for high-resolution spectra, at which a spectral resolution of 0.45 eV can be achieved. The source and analyser were installed at the magic angle. The angle of emission was 0° with respect to the surface normal. The data were evaluated using CasaXPS v2.3.25 using Scofield sensitivity factors. The uncertainties in the elemental concentrations was estimated using the Monte Carlo method as implemented in the latest CasaXPS versions for the region quantification (see the button “Calculate error bars” in the region quantification property page). The binding energy scale was referenced to the main C1s line at 285.0 eV. Mixed Gaussian/Lorentzian line shapes (70/30) were employed for fitting the high-resolution spectra.

Sputtering was performed by gas cluster ion bombardment (GCIB) using Ar clusters with 500 atoms at an energy of 8000 eV. The beam was scanned across 2.5 mm × 2.5 mm at a cycle time of 3 s. The sputter rate was 1.8 nm s^−1^ as determined by sputtering a spin-coated PMMA reference.

## 3. Results and Discussion

**Figure 1:**
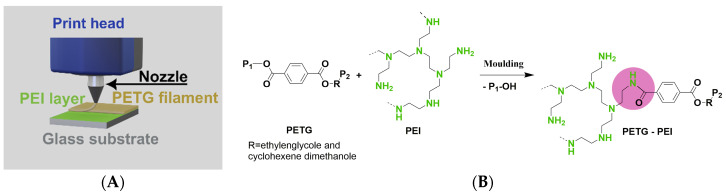
(**A**) Scheme of sample preparation by moulding. (**B**) Proposed grafting reaction of PEI onto PETG, whereby PEI is bound to PETG by an amide group (red circle). P_1_, P_2_ = residues of the PETG chain, R = ethyleneglycol and cyclohexene dimethanol.

PEI of three different molar masses were grafted to PETG: PEI-750k (750 kg mol^−1^), PEI-25k (25 kg mol^−1^), and PEI-2k (2 kg mol^−1^). Investigations started with the largest molecule PEI-750k. The PEI-coated glass slides were placed in the recess of the print bed. A PETG part was then printed on top of that coated area as shown in Figure 1A. We had investigated those coupling reactions in advance by molecular simulations, model reactions, and injection moulding experiments [41,42]. We know from previous research that only the chemical groups on the outermost surface layer of the functional polymer layer can react with the ester groups on the surface of the polymer melt. There is no mixing within the very small time until the interphase cools down and the materials freeze. That is why the thickness of the functional polymer layer is not crucial, as long as a complete coverage is realised. Excess PEI was removed after printing by rinsing the PETG parts in water, which is a good and selective solvent for PEI, for 1 h. This procedure ensured that no unbound PEI dissolved from the PETG surface during further treatment of the part. After drying, the surfaces were very hydrophilic and could be wetted by water. However, contact angles could not be measured due to structure formation on the surface as a result of the preparation method.

### 3.1. Grafting Reaction

The surface chemistry of the PETG parts after printing on different PEI coatings and washing with water was investigated by XPS. Table 1 shows the XPS elemental composition observed on these surfaces. The data were complemented by a sputtered cleaned sample of PEI-2k using an Ar cluster ion gun (Thermo Fisher, Waltham, MA, USA), which was assumed to resemble the surface of the unmodified PETG. This approach was chosen over the direct analysis of unmodified PETG, because artefacts of potential additive segregates may be thereby avoided.

All analysed surfaces were characterized by the presence of C, O, N, and Cl, which was basically in line with the composition of the PETG parts and the PEI used. The observed Cl, which was present especially after using PEI-25k, was believed to relate to adsorbed HCl contaminations in view of the absence of other heteroatoms.

However, the results clearly show increasing N concentrations with the use of increasingly higher molar mass of PEI. The N concentrations range from around 6.4 (PEI-2k) up to 23.5 at% (PEI-750k). This shows that PEI residues are immobilized on the surface of the PETG. The loading of these deposits is primarily determined by their molar mass.

The PEI layer thicknesses on PETG surfaces were estimated by different approaches for a basic characterization of the coverage. First, the layer thickness of the PEI was estimated using the experimental N concentrations and Hill’s equation. This approach was based on the assumption of a homogeneous PEI layer covering the PETG. Given an effective attenuation length of 3 nm, the layer thicknesses were in the range of around 0.6, 2.0 and 3.7 nm (see Table 1).

For comparison, the expected thickness of completely closed PEI monolayers was also calculated using their number average molar mass (1.8, 10, 60 kg mol^−1^) combined with the density of the material, which was 1.03 g cm^−3^. This resulted in thickness values of around 1.4, 3.4, and 4.6 nm for the different molar masses employed.

The comparison of all the estimated thickness values shows that the surface modification yields more or less monolayer deposits of PEI on the PETG surfaces, while there may be a tendency for a sub-monolayer coverage.

**Figure 2:**
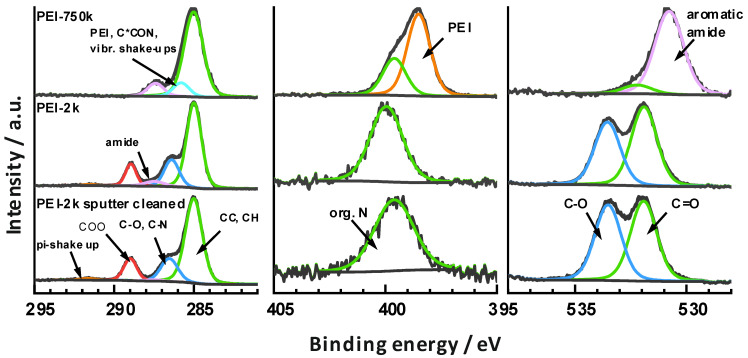
High-resolution XPS core level spectra of the modified and sputter cleaned PETG surfaces of PEI-2k and PEI-750k. C*CON refers to the carbon in alpha-position to the amide group.

The high-resolution core level spectra and peak fits are shown in Figure 2.

**Sputter cleaned PEI-2k sample:** The C1s spectrum of the sputter cleaned PETG surface made from a PEI-2k sample showed a line shape that compares well with data in the literature on PET type polymers [47]. The chemical components of the spectral fit were assigned accordingly, based on their binding energies to aliphatic/aromatic CC and CH at 285.0 eV, to alcohols C-O and amines C-N at 286.5 eV, to ester COO at 299.0 eV, and to the π-shake up at around 291.6 eV.

The O1s spectrum was characterized by the presence of two components at 531.9 (C=O) and 533.2 eV (C-O), which were ascribed to the ester and alcohol groups in the material.

The analysis also revealed still the presence of a low amount of a single N1s component at 399.6 eV. This was ascribed to organic N components within the material and potentially also to PEI residues, because the surface was slightly corrugated, so some regions may have been shadowed during sputtering.

**PEI-2k:** The PETG surface after modification with PEI-2k showed spectra quite similar to what was observed for the sputter cleaned PETG. However, another component was identified in the C1s at 287.5 eV (5%) and was ascribed to newly formed amide groups. This assignment is based on the XPS analysis of aramide fibres by de Lange et al. [48], who observed this component at 288.0 eV. The shift of 0.5 eV in our case may be the consequence of differences in the binding energy scale reference. This is because a mixed aromatic/aliphatic main component was expected in the present case, while the aramide fibres showed up a basically clean aromatic C1s main component so that a higher chemical shift was expected there (around 0.2–0.5 eV is reasonable according to the data provided by Beamson and Briggs [47]). Consequently, a grafting reaction as shown in Figure 1B was carried out.

The interpretation of the spectral data in terms of newly formed amide groups is also consistent with the other core level spectra. The N1s component shifted to 400.0 eV, which is typical for a variety of organic N compounds including aromatic amides [48]. However, the O1s showed a slight decrease in the C-O signal and thereby indicated decreased concentrations of alcohol and ester groups. This highlighted that the alcohols released by the amidation were mostly removed from the surface within the subsequent washing step. A stoichiometric calculation supported this hypothesis: taking the intensity fraction of the C1s amide signal (5%) and the total C concentration, one ended up at 3.5 at% of amide bound carbon. Similarly, the intensity of the O1s C-O signal decreased by 11%, which corresponds to 2.8 at% of alcohol bound oxygen. That is, the amide concentration increased nearly quantitatively with the loss of C-O groups, which would be expected for an amidation reaction.

**PEI-750k:** The C1s line shape after modification with PEI-750k was totally changed and simplified to basically three chemical components at 285.0 eV (CC, CH) and 287.4 eV (aromatic amide). The third component was observed at 285.8 eV and only barely separated from the main peak. Owing to the low chemical shift, a range of assignments was possible including not only the amides C-N groups, but also vibrational shake-ups of C-H groups, the secondary carbon to the amide groups C*CON, and the CH_2_ groups of PEI. It was noted that the binding energies of the C1s and N1s signals of PEI are rather exotic and commonly found at 285.5 and 399.0 eV. However, the C1s clearly showed a quantitative transformation of all ester groups into amides within the complete depth of information, which was around 10 nm.

The O1s line shape also showed a drastic change and the formation of basically a single peak at 530.8 eV. This low binding energy is rare for organic compounds, but agreed well with the O1s of aramide fibres at 530.8 eV [48].

The N1s instead was characterized by the presence of two components at 399.6 eV (31%) and 398.5 eV (69%). According to their binding energies, the latter was ascribed to PEI and the former to aromatic amides. Consequently, about 31% of the amino groups were converted to amide bonds to PETG. Again, slight deviations of around 0.4 eV were observed when comparing with the literature. This may again be a consequence of an inaccurate definition of the binding energy scale, because the main C1s component was not related to typical aliphatic groups, but rather to a mixture of aromatic signals combined with the PEI backbone signals, which were both shifted. That is, if one would reference the main line to 285.5 eV, which was the binding energy found for PEI, all discrepancies would be resolved. However, this procedure would be as artificial as the conventional procedure and was therefore omitted.

The observed intensity fractions of the N1s components can be well rationalized when considering the quantitative nature of the amidation reaction. The amount of reactive primary amines of hyper-branched PEI is typically around 30%, and this matches the fractional amount of amides found.

However, the complete amidation of the uppermost 10 nm was surprising, because the nominal layer thickness of the PEI deposits was lower. Apparently, a new amide-containing phase was formed at the near-surface region. Considering that high molar mass polymer combinations are usually immiscible, it is suggested that the chemical reaction between the polyester and polyamine leads to a reactive interdiffusion of the materials at the nanoscale. This does not require a complete dissolution and intermingling of the different polymers within another but rather results in a dispersion of PEI molecules within a modified PETG matrix. Similar results were found by Monte-Carlo simulations of the interface between two reactive immiscible polymers using the bond fluctuation model [44].

### 3.2. Adsorption of PdCl_2_

A PETG part prepared with grafted PEI-750k that was rinsed in PdCl_2_ solution in 0.1 M hydrochloride acid, exhibited the typical elements C and O of the PETG filament, see Table 2. Ti stems from the TiO_2_ pigment used for white pigmentation. The method is not sensitive for N. The Pd ions were, obviously, attached to the PEI layer by coordinative bonds to the amino groups [49,50]. The stability constant of the Pd-Amine chelate is exceptionally high, so that a formation occurs also in high acidic medium.

The chlorine ions were probably the counter ions of Pd ions in this complex, but adding of HCl to amino groups under the formation of an organic ammonium chloride may also be possible. Nevertheless, the adsorption of Pd and Cl pointed to a grafted PEI layer on the PETG surface that still exhibited the function of amino groups. The elemental mappings in Table 2 show smooth distributions of C and O over the surface of a printed strand. Moreover, the distributions of Cl and Pd were smooth, pointing to a flat film of PEI grafted to the PETG surface.

### 3.3. Adsorption and Desorption of Anionic Dye

**Figure 3:**
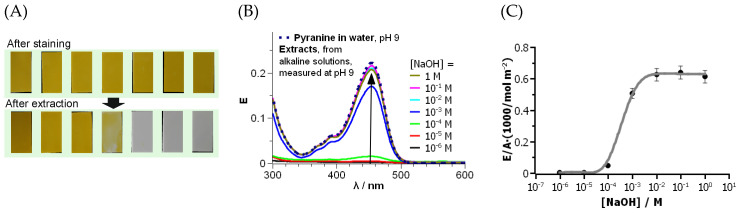
Results of the pyranine adsorption on PETG surfaces grafted with PEI-750k. (**A**) Photographs of stained PETG-PEI-750k parts after staining with pyranine and removing of unbound pyranine (**top**), and after extraction in NaOH solutions of different concentrations from left to right: 10^−6^ M, 10^−5^ M, 10^−4^ M, 10^−3^ M, 10^−2^ M, 10^−1^ M, and 1 M (**bottom**). Corresponding parts are placed in the same order top and bottom. (**B**) Optical spectra of the extract solutions with different NaOH concentrations. The spectra are normalised to the same volume of the extract of 50 mL. A spectrum (dotted line) of pyranine in water at pH 9 is added as reference, [Pyranine] = 1.25 × 10^−5^ M. (**C**) Amount of pyranine per PETG part surface area, calculated from absorption maxima of the extract solution and the printed area as a function of NaOH concentration in the extract solution. The grey line is a guide to the eyes.

Staining was performed by dipping PETG parts in an alkaline pyranine solution. The parts were then rinsed in water to remove unbound pyranine. The photographs in Figure 3A, top row, show deep and uniform pyranine staining over all the PETG surfaces. Reference parts printed on clean glass slides (without PEI) did not stain, i.e., staining with pyranine was selective for PETG parts modified with PEI. The adsorption of pyranine on the PETG surfaces grafted with PEI-750k was strong, and pyranine did not desorb in water, which is actually a good solvent for pyranine. Tests revealed that desorption had not occurred even after several days. A probable reason for that was electrostatic attraction between the high number of partly positively charged amino groups of PEI and the three negatively charged sulfonate groups per pyranine molecule.

### 3.4. Behaviour in Alkaline Medium

#### 3.4.1. Extraction in Alkaline Medium

The stained PETG parts were rinsed in NaOH solutions of different concentrations for 1 h. The pH values of the resulting extracts were adjusted to 9. Finally, the volumes of the extracts were equalled with buffer (pH value of 9) solution to 50 mL. The solution of the extract with 1 M NaOH was diluted to 100 mL, but its extinctions were normalised to 50 mL for comparison with the other spectra. Optical spectra of the extract solutions were recorded from 300 to 600 nm to quantify the extracted amount of pyranine. If necessary, a baseline correction was performed. Raw data are included in Table S2-1.

No pyranine desorption took place in neutral water, according to an optical evaluation of the samples and the extracts. Figure 3A, bottom row, shows the stained PETG part surfaces after extraction in NaOH solutions of different concentrations. The photographs of the parts extracted in 10^−6^ to 10^−4^ M NaOH solution exhibited a yellow colour similar to the images of the PETG parts after staining on the top row. The PETG sample extracted in 10^−3^ M NaOH showed a decreased staining intensity. A higher concentration of NaOH extraction solution resulted in completely decolourised samples.

Figure 3B shows the optical spectra of the extract solutions. All spectra are qualitatively similar to that of the freshly prepared solution of pyranine (dashed line) at the same pH value as in the extracts. This proves that pyranine did not undergo chemical transformation during the extraction procedure. However, the absorption maxima of the solutions were different. The amount of pyranine per sample area in Figure 3C was calculated according to Lambert–Beers law and the volume of the extract solution. The amount was normalised to the surface area of the sample and related to the NaOH concentration in the extract solution. The results confirmed the conclusions drawn from the photographs: No optical absorptions were observed in the solutions extracted with NaOH in the concentration range from 10^−6^ to 10^−4^, i.e., no desorption of pyranine took place here. A partial desorption of pyranine took place at 10^−3^ M NaOH. Pyranine completely desorbed from the PETG parts at higher NaOH concentrations in accordance with the photographs in Figure 3A. The maximum amount of adsorbed pyranine was about 7 × 10^−4^ mol m^−2^. From steric considerations, this corresponds to more than one pyranine molecule per molecular area, i.e., several molecules must have been adsorbed on top of each other within the PEI layer. This finding demonstrates the large modification effect of the grafting procedure.

It is known that the zeta potential of PEI becomes negative at high pH values [51]. The desorption of the negatively charged pyranine is, consequently, a result of reversing the surface charge of the PEI-modified PETG surface and of electrostatic repulsion between PEI and pyranine. However, the data cannot exclude completely any desorption of PEI at this stage.

#### 3.4.2. Stability of Grafting

The previous experiments revealed that PEI-750k was grafted onto the PETG surface, and the grafting layer withstood an extraction in water and the different staining procedures. The PEI layer was quite thick, as it adsorbed a large amount of pyranine. This chapter investigates the adsorption capacity of PEI of different molar masses for several extraction cycles in 0.01 M NaOH solution. In a second investigation, several extraction cycles were carried out with NaOH solutions of different concentrations. Raw data are included in Tables S3-1–S3-3, S4-1 and S4-2.

**Figure 4:**
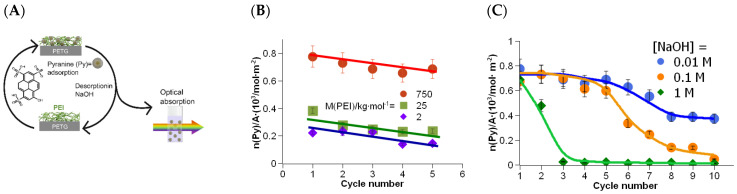
Staining-extraction cycles with PEI of different molar masses. (**A**) Scheme of the experimental procedure. A PETG part, grafted with PEI (different molar masses) is rinsed in pyranine solution, resulting in a stained PETG surface. The part is then rinsed in NaOH solution (different concentrations), resulting in desorption of pyranine. The amount of pyranine in the extract is measured by optical absorption. (**B**) Absorption maxima of extraction solutions for different molar masses of PEI and for multiple staining-extraction cycles. Extraction solution: [NaOH] = 10^−2^ M. The lines should merely illustrate a trend for the reader. (**C**) Adsorbed amount of pyranine after multiple staining-extraction cycles in different alkaline concentrations as an approximation for the amount of PEI-750k bound to the PETG surface.

The grafting procedure was carried out with PEI-750k, PEI-25k, and PEI-2k. The amount of adsorbed pyranine was measured by extraction in 10^−2^ M NaOH solution, as described in the previous section. However, samples were re-stained after pyranine extraction here, and the amount of adsorbed pyranine in the next cycle was measured again in the extract. Those staining-extraction cycles (see Figure 4A) were repeated several times.

The high molar mass PEI-750k exhibited an adsorbed amount of pyranine of about 8 × 10^−4^ mol∙m^−2^. It was somewhat reduced in the second cycle, pointing to the removal of PEI during the extraction in alkaline solution. Increasing the cycle number decreased the adsorbed amount almost linearly, as the trend line shows in the Figure 4B. However, the adsorbed amount after 5 cycles is still high, pointing to a robust grafting of PEI onto the PETG surface.

The adsorbed amount of pyranine for PEI-25k was about 4 × 10^−4^ mol m^−2^. This is about half the value of that of the high molar mass PEI-750k. The relation fits well with the ratio of the thickness of both PEI types found in the XPS investigation, see Figure 2A. With increasing cycle number, the adsorbed amount of pyranine decreased significantly, too. This trend continued with the low molar mass PEI-2k. Again, the adsorbed amount of pyranine in the first cycle was lower with a value of about 2 × 10^−4^ mol m^−2^. Even for PEI with the lowest molar mass, the amount of adsorbed pyranine and, thus, the amount of grafted PEI on the PETG surface, was still high. Remarkably, the trend lines of all three molar masses of PEI showed approximately the same rise. This suggests that the loss of adsorption capacity is not a function of the average molar mass.

In the previous section, we demonstrated the grafting of PEI of different molar masses and its robust bonding on the PETG surface. However, repeated extraction cycles obviously removed some of the grafted PEI. This section deals with investigations on the reasons for the decreasing adsorbed amount of pyranine.

Three reasons for this are discussed in the following. First, it might be possible that the first extraction in water after the moulding procedure did not remove the entire unbound PEI. One would assume that this is independent of the NaOH concentration. Second, the amide bond formed during grafting may be decomposed. This decomposition is not very likely, because the amide bond is usually resistant against alkali. The third reason could be a decomposition of PETG. PETG, like any polyester, undergoes a saponification reaction in an alkaline medium. Hydrolysis of an ester bond adjacent or in vicinity to an amide bond could fragment the PETG chain. Saponification in vicinity of a high number of amide bonds per PEI molecule could result in releasing and dissolution of the PEI molecule with residues of PETG attached to the amide bonds.

In the following experiments, the staining-extraction experiments were carried out with alkali solutions of different extraction concentrations. Since desorption of pyranine was only effective above a NaOH concentration of 10^−2^ M, only the concentrations of 10^−2^, 10^−1^, and 1 M were used.

The adsorbed amount of pyranine in the first extract with 10^−2^ M NaOH was again about 8 × 10^−4^ mol m^−2^. The value decreased approximately linear during the first extractions (see Figure 4C), as already shown in the previous experiment. However, after cycle number 5, the adsorbed amount of pyranine declined. A constant amount was established after cycle number 8. Desorption of PEI was, probably, a result of decomposition of PETG by saponification.

The grafting of PEI was also stable against 10^−1^ M NaOH, at least up to cycle number 3, followed by continuous decrease in the adsorbed amount of pyranine to almost zero, i.e., the amount of bonded PEI decreased during the procedure, and finally all PEI was removed from the PETG surface. The decrease in adsorbed amount of pyranine was even higher at an NaOH concentration of 1 M, pointing to fast and effective saponification of PETG.

## 4. Conclusions

In this work, a surface-grafting approach was investigated to graft a functional polymer layer onto thermoplastics during moulding. PEI of different molar masses were successfully transferred onto surfaces of PETG parts using an FFF process. XPS investigations showed that the polyamine was grafted on the surface of the polyester with a very high density of amino groups per area and an even distribution over the surface. Chemical bonding was achieved by amide bonds, formed by reactions between the amino groups of the PEI and ester groups of the PETG. The thickness of the grafted polyamine and, thus, the grafting effect, were very high and depended on the molar mass of the functional molecule. The highest effect was established with the highest molar mass of PEI (750 kg mol^−1^). The thickness of the grafted PEI was 3.7 nm. The distribution over the surface was even. The adsorption capacity for pyranine was 7 × 10^−4^ mol m^−2^.

This type of reaction requires typically a catalyst. However, the reaction conditions at the surface of a melt during moulding supplied a high temperature, which enables grafting without an external catalyst. Despite the absence of a catalyst and the very short reaction time, the conversion of amino groups to amide bonds was about 30%. That is optimal, because it ensures a stable grafting as well as remaining of many amino groups for surface functionalisation.

Robustness of the PEI grafting was verified by staining the PETG parts with a pyranine dye followed by extraction in alkaline solutions of various concentrations. Grafting was stable in neutral water and also in an alkaline medium as long as the concentration of NaOH did not exceed 10^−3^ M. At higher alkaline concentrations, the formed amide bonds were usually still stable, but saponification of ester groups within the polyester chain segments on the surface occurred. This resulted in releasing of PETG fragments together with previously grafted polyamine. This decomposition of the PETG surface in alkaline medium is usually not visible macroscopically. Thus, the grafting and staining procedure may also be used for assessment of the stability of a polyester surface in a certain environment.

The grafting procedure using FFF is suitable for producing low numbers of polymer samples with selective surface functionalization by amino groups. Manufacturing could be conducted in any chemical or bio-chemical laboratory to produce aminated polyester surfaces on demand. For large-scale production, industrial processes such as injection moulding can be applied.

The advantage of this surface grafting approach is the easy and effective one-step preparation procedure. Any other polyester surface may also be modified using this reaction, e.g., polyethylene terephthalate, polybutylene terephthalate, polylactide, polycarbonate, thermoplastic polyurethanes with polyester segments, or blends with these polyesters. Thus, the approach may be very versatile for surface functionalisation.

Moreover, the aminated polyester surface could be used as a precursor to attach further molecules by selective bonding, e.g., by epoxides or isocyanates. In addition, proteins or metal ions could be immobilized on the surface by coordinative bonds, like Cu^2+^.

## Figures and Tables

**Table 1:** Elemental surface compositions by XPS.

M_W_(PEI)/ kg mol^−1^	Sputter Time/s	C		O		N		Cl		d(PEI)/nm
		at%	STD/%	at%	STD/%	at%	STD/%	at%	STD/%	
2	0	69.5	0.4	23.9	0.3	6.4	0.3	0.19	0.05	0.6
	3	71.7	0.4	24.8	0.4	3.5	0.3	0.12	0.05	
	6	72.4	0.4	24.3	0.3	3.2	0.3	0.03	0.05	
	9	71.8	0.4	25.6	0.4	2.6	0.3	0.02	0.05	
25	0	65.7	0.2	15.1	0.2	16.3	0.2	2.92	0.05	2.0
750	0	65.8	0.2	10.5	0.2	23.5	0.2	0.16	0.02	3.7

**Table 2:** Elemental composition of the surface layer of a printed PETG sample grafted with PEI-750k, rinsed in PdCl_2_ solution, by EDX.

Name	Carbon	Oxygen	Chlorine	Palladium	Titan
Symbol	C	O	Cl	Pd	Ti
Colour					-
Atomic Concentration	73.857	21.673	3.022	1.231	0.217
Mapping graph	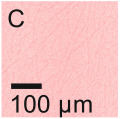	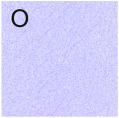	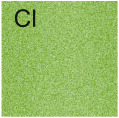	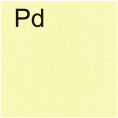	-

## Data Availability

Data are contained within the article and Supplementary Materials.

## Supplementary Materials

The following supporting information can be downloaded at: https://www.mdpi.com/article/10.3390/polym16050644/s1, Figure S1-1. Extinction as a function of pyranine concentration. Figure S1-2. Photographs of seven printed samples. Table S1-1. Evaluation of the statistical error of the measurement procedure. Table S2-1. Measurement data. Table S3-1. Pyranine amounts for PEI-750k extracted with [NaOH] = 0.01 M. Table S3-2. Pyranine amounts for PEI-750k extracted with [NaOH] = 0.1 M. Table S3-3. Pyranine amounts for PEI-750k extracted with [NaOH] = 1 M. Table S4-1. Pyranine amounts for M_w_(PEI) = 25 kg mol^−1^. Table S4-2. Pyranine amounts for M_w_(PEI) = 2 kg mol^−1^. Reference [52] is cited in the Supplementary Materials.

## References

[1] Hasan A., Pandey L.M. (2015). Review: Polymers, Surface-Modified Polymers, and Self Assembled Monolayers as Surface-Modifying Agents for Biomaterials. Polym.-Plast. Technol. Eng..

[2] Hoffman A. (1995). Surface Modification of Polymers. Chin. J. Polym. Sci..

[3] Govindarajan T., Shandas R. (2014). A Survey of Surface Modification Techniques for Next-Generation Shape Memory Polymer Stent Devices. Polymers.

[4] Gao W., Han X., Li Y., Zhou Z., Wang J., Shi R., Jiao J., Qi Y., Zhou Y., Zhao J. (2022). Modification Strategies for Improving Antibacterial Properties of Polyetheretherketone. J. Appl. Polym. Sci..

[5] Moad G. (1999). The Synthesis of Polyolefin Graft Copolymers by Reactive Extrusion. Prog. Polym. Sci..

[6] Ratzsch M., Arnold M., Borsig E., Bucka H., Reichelt N. (2002). Radical Reactions on Polypropylene in the Solid State. Prog. Polym. Sci..

[7] Foerster F. (2022). Atmospheric Pressure Plasma in Industrial Applications: Surface Treatment of Thermally Sensitive Polymers. Plasma Process. Polym..

[8] Siow K.S., Britcher L., Kumar S., Griesser H.J. (2006). Plasma Methods for the Generation of Chemically Reactive Surfaces for Biomolecule Immobilization and Cell Colonization—A Review. Plasma Process. Polym..

[9] Arpagaus C., Oberbossel G., von Rohr P.R. (2018). Plasma Treatment of Polymer Powders—from Laboratory Research to Industrial Application. Plasma Process. Polym..

[10] Tengsuthiwat J., Sanjay M.R., Siengchin S., Pruncu C. (2020). 3D-MID Technology for Surface Modification of Polymer-Based Composites: A Comprehensive Review. Polymers.

[11] Rasal R.M., Janorkar A.V., Hirt D.E. (2010). Poly(Lactic Acid) Modifications. Prog. Polym. Sci..

[12] Pillai R.R., Thomas V. (2023). Plasma Surface Engineering of Natural and Sustainable Polymeric Derivatives and Their Potential Applications. Polymers.

[13] Omran A.V., Baitukha A., Pulpytel J., Sohbatzadeh F., Arefi-Khonsari F. (2018). Atmospheric Pressure Surface Modification and Cross-Linking of UHMWPE Film and inside HDPE Tube by Transporting Discharge. Plasma Process. Polym..

[14] Morent R., De Geyter N., Desmet T., Dubruel P., Leys C. (2011). Plasma Surface Modification of Biodegradable Polymers: A Review. Plasma Process. Polym..

[15] Khulbe K.C., Feng C., Matsuura T. (2010). The Art of Surface Modification of Synthetic Polymeric Membranes. J. Appl. Polym. Sci..

[16] Friedrich J. (2011). Mechanisms of Plasma Polymerization—Reviewed from a Chemical Point of View. Plasma Process. Polym..

[17] Grundmeier G., von Keudell A., de los Arcos T. (2015). Fundamentals and Applications of Reflection FTIR Spectroscopy for the Analysis of Plasma Processes at Materials Interfaces. Plasma Process. Polym..

[18] Decher G., Hong J.-D. (1991). Buildup of Ultrathin Multilayer Films by a Self-Assembly Process, 1 Consecutive Adsorption of Anionic and Cationic Bipolar Amphiphiles on Charged Surfaces. Makromolekulare Chemie. Macromol. Symp..

[19] Witt M.A., Valenga F., Blell R., Dotto M.E.R., Bechtold I.H., Felix O., Pires A.T.N., Decher G. (2012). Layer-by-Layer Assembled Films Composed of “Charge Matched” and “Length Matched” Polysaccharides: Self-Patterning and Unexpected Effects of the Degree of Polymerization. Biointerphases.

[20] Gill R., Mazhar M., Felix O., Decher G. (2010). Covalent Layer-by-Layer Assembly and Solvent Memory of Multilayer Films from Homobifunctional Poly(Dimethylsiloxane). Angew. Chem.-Int. Edit..

[21] Petzold G., Schwarz S., Dutschk V. (2014). Polyelectrolyte-Surfactant Complexes and Their Influence on the Wettability of Different Polymer Surfaces. Colloid Polym. Sci..

[22] Galvin C.J., Genzer J. (2012). Applications of Surface-Grafted Macromolecules Derived from Post-Polymerization Modification Reactions. Prog. Polym. Sci..

[23] Jo H., Theato P., Vana P. (2016). Post-Polymerization Modification of Surface-Bound Polymers. Controlled Radical Polymerization at and from Solid Surfaces.

[24] Henze M., Maedge D., Prucker O., Ruehe J. (2014). “Grafting Through”: Mechanistic Aspects of Radical Polymerization Reactions with Surface-Attached Monomers. Macromolecules.

[25] Prucker O., Ruhe J. (1998). Synthesis of Poly(Styrene) Monolayers Attached to High Surface Area Silica Gels through Self-Assembled Monolayers of Azo Initiators. Macromolecules.

[26] Ruhe J., Ballauff M., Biesalski M., Dziezok P., Grohn F., Johannsmann D., Houbenov N., Hugenberg N., Konradi R., Minko S., Schmidt M. (2004). Polyelectrolyte Brushes. Polyelectrolytes with Defined Molecular Architecture I.

[27] Ruhe J. (1998). Polymers Grafted from Solid Surfaces. Macromol. Symp..

[28] Edmondson S., Osborne V.L., Huck W.T.S. (2004). Polymer Brushes via Surface-Initiated Polymerizations. Chem. Soc. Rev..

[29] Zdyrko B., Luzinov I. (2011). Polymer Brushes by the “Grafting to” Method. Macromol. Rapid Commun..

[30] Varvarenko S., Samaryk V., Nosova N., Puzko N., Taras R., Tarnavchyk I., Voronov A., Kohut A., Voronov S., Mormann W. (2010). Prediction of Interfacial Interactions between Polymer Layers. Proceedings of the Polychar-18 World Forum on Advanced Materials.

[31] Zhao B., Brittain W.J. (2000). Polymer Brushes: Surface-Immobilized Macromolecules. Prog. Polym. Sci..

[32] Moulay S. (2018). Functionalized Polystyrene and Polystyrene-Containing Material Platforms for Various Applications. Polym.-Plast. Technol. Eng..

[33] Kabanov V. (1995). Radiation-Induced Graft-Polymerization as a Method for Surface Modification of Polymers. Vysokomol. Soedin..

[34] Wang J., Chen H. (2023). Macromolecular Modification Strategies for Biomaterial Surface: Challenges in Fundamental Research and Applications. Macromolecules.

[35] Akimoto A.M., Ohta Y., Koizumi Y., Ishii T., Endo M., Enomoto T., Nishimoto T., Yoshida R. (2023). A Surface-Grafted Hydrogel Demonstrating Thermoresponsive Adhesive Strength Change. Soft Matter.

[36] Kato K., Uchida E., Kang E.T., Uyama Y., Ikada Y. (2003). Polymer Surface with Graft Chains. Prog. Polym. Sci..

[37] Park H., Wiesing M., Zimmermann P., Janke A., Schwarz S., Nagel J. (2022). Laser-Assisted Direct Grafting of Poly(Ethyleneimine) on Poly(Methyl Methacrylate). Polymers.

[38] Nasef M.M., Gupta B., Shameli K., Verma C., Ali R.R., Ting T.M. (2021). Engineered Bioactive Polymeric Surfaces by Radiation Induced Graft Copolymerization: Strategies and Applications. Polymers.

[39] Nagel J., Brauer M., Hupfer B., Grundke K., Schwarz S., Lehmann D. (2004). Investigations on the Reactive Surface Modification of Polycarbonate by Surface-Reactive Injection Molding. J. Appl. Polym. Sci..

[40] Nagel J., Zimmermann P., Schubert O., Simon F., Schlenstedt K. (2017). Coupling of Carboxylic Groups onto the Surface of Polystyrene Parts during Fused Filament Fabrication. Appl. Surf. Sci..

[41] Nagel J., Zimmermann P., Schwarz S., Schlenstedt K. (2018). Selective Grafting of Polyamines to Polyether Ether Ketone Surface during Molding and Its Use for Chemical Plating. Coatings.

[42] Nagel J., Heinrich G. (2012). Temperature Transitions on the Surface of a Thermoplastic Melt during Injection Moulding and Its Use for Chemical Reactions. Int. J. Heat Mass Transf..

[43] Deutsch H., Binder K. (1991). Interdiffusion and Self-Diffusion in Polymer Mixtures—A Monte-Carlo Study. J. Chem. Phys..

[44] He X.H., Nagel J., Lehmann D., Heinrich G. (2005). Interface Structure between Immiscible Reactive Polymers under Transreaction: A Monte Carlo Simulation. Macromol. Theory Simul..

[45] Zimmermann P., Schlenstedt K., Schwarz S., Vehlow D., Blanke M., Fery A., Nagel J. (2022). Green Approach for Manufacturing of Polymer Surface Structures with Microcavities Having Robust Chemically Functionalized Inner Surfaces. ACS Appl. Polym. Mater..

[46] Fakirov S. (1999). Transreactions in Condensation Polymers.

[47] Briggs D., Beamson G. (2000). XPS of Polymers Database.

[48] de Lange P.J., Akker P.G., Maas A.J.H., Knoester A., Brongersma H.H. (2001). Adhesion Activation of Twaron® Aramid Fibres Studied with Low-Energy Ion Scattering and x-Ray Photoelectron Spectroscopy. Surf. Interface Anal..

[49] Jin H., Yang J., Jun-Feng Z., Wei-Jiang H. (2009). Intermolecular Interaction between Pd-II Complex of 2-(Bis(2-aminoethyl)amino)ethanol and Met, His or Cys-containing Peptides. Chin. J. Inorg. Chem..

[50] Ribaudo F., van Leeuwen P.W.N.M., Reek J.N.H. (2009). Phosphorus Functionalized Dendrimers and Hyperbranched Polymers: Is There a Need for Perfect Dendrimers in Catalysis?. Isr. J. Chem..

[51] Bucatariu F., Ghiorghita C.-A., Dragan E.S. (2018). Cross-Linked Multilayer Films Deposited onto Silica Microparticles with Tunable Selectivity for Anionic Dyes. Colloids Surf. A Physicochem. Eng. Asp..

[52] Lindsey J.S., Taniguchi M., Du H. PhotochemCAD 1998–2023. https://photochemcad.com/databases/common-compounds/polycyclic-aromatic-hydrocarbons/pyranine.

