# Supplementary material for: Efficient Approach for Direct Robust Surface Grafting of Polyethyleneimine onto a Polyester Surface during Moulding
Source: Polymers (Basel). 2024 Feb 27;16(5):644. doi: 10.3390/polym16050644 (PMC10933935; doi:10.3390/polym16050644)
Supplement: Supplementary file 1 [file polymers-16-00644-s001.zip › polymers-2849943-supplementary.pdf]

Efficient approach for direct robust surface grafting of polyethylenimine onto a polyester surface during moulding

Philipp Zimmermann[1], Silven Frohs[1], Martin Wiesing[2], Kamal Meena[1], Jürgen Nagel[1]

Supporting Information

S1) General conditions

a) Evaluation of extinction coefficient of pyranine over broad concentration range

A stock solution of pyranine with a concentration of 10^{-4} M in water was prepared and its pH value adjusted to 9 by adding 1 M NaOH solution and borate buffer. Solutions of this stock solution were prepared by dilution with borate puffer at pH value of 9. Optical spectra were recorded with a Cary 60 spectrometer (Agilent, USA) from 300 to 600 nm. The maxima absorption was at 455 nm for all solutions. The maxima were displayed as a function of concentration in Figure S1-1. An extinction coefficient of 20 950 l mole^{-1} cm^{-1} was calculated by linear regression over the concentration range from 10^{-6} to 10^{-4} M. This equals the value mentioned in literature [52].

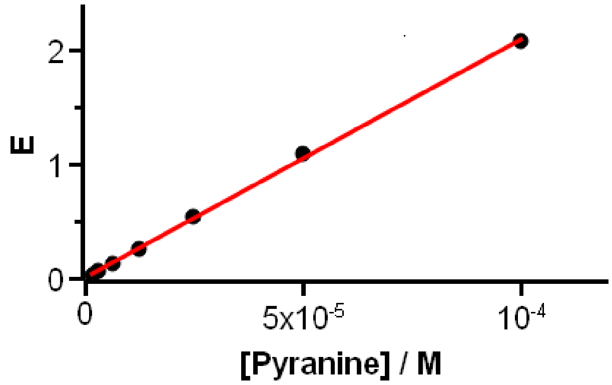

Figure S1-1. *Extinction as a function of pyranine concentration.* $\varepsilon(\lambda = 455nm) = 20950$ *l mol*$^{-1}$ *cm*$^{-1}$

b) Error evaluation

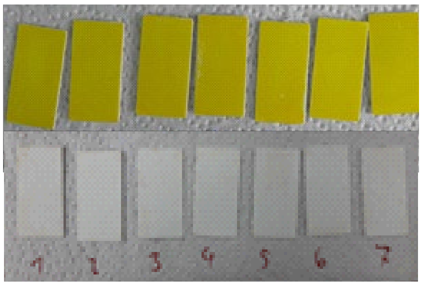

Figure S1-2. *Seven printed samples, extracted in water and stained with pyranine (top) and after extraction in 1 M NaOH solution (bottom).*

Seven samples were prepared and treated according to the experimental procedure under identical conditions. The average and the relative standard deviation were calculated.

Table S1-1. Evaluation of the statistical error of the measurement procedure.

**Sample no.**	**E (455 nm)**	**v^a/ml**	**n^b(Py)/nmol**	**n/A^c(Py) / mol m^{-2}**
**1**	0.285	50	680	$0.85 \cdot 10^{-3}$
**2**	0.25	50	595	$0.744 \cdot 10^{-3}$
**3**	0.245	50	570	$0.713 \cdot 10^{-3}$
**4**	0.245	50	585	$0.731 \cdot 10^{-3}$
**5**	0.315	50	740	$0.925 \cdot 10^{-3}$
**6**	0.235	50	560	$0.70 \cdot 10^{-3}$
**7**	0.135	100	644	$0.805 \cdot 10^{-3}$
Average				*$0.78 \cdot 10^{-3}$*
deviation				*$0.77 \cdot 10^{-3}$*
relative deviation /%				*10*

a) Volume of the extract solution after dilution and adjustment of pH value to 9.

b) Amount of pyranine in extract solution according to Lambert's law.

c) Amount of pyranine of (b) divided by sample area.

Conclusion: The error of the measurement procedure was about 10 %. Extractions were made with 1 M NaOH. M_w(PEI)=750 kg mol^{-1}.

S2) Variation of alkali concentration

The samples prepared using PEI with different molar masses were stained with pyranine and extracted in different concentrations of NaOH. The raw data presented here correspond to the appropriate experiments in the main part of the manuscript.

Table S2-1. *Measurement data.* $M_w(PEI)=750\ kg\ mol^{-1}$.

Sample no.	[NaOH]/M	E(455 nm)	v/ml	n/A / mol·m^{-2}
**1**	10^{-6}	0.0004	50	$0.0119 \cdot 10^{-4}$
**2**	10^{-5}	0.002	50	$0.0596 \cdot 10^{-4}$
**3**	10^{-4}	0.001	50	$0.0298 \cdot 10^{-4}$
**4**	10^{-3}	0.16	50	$4.7681 \cdot 10^{-4}$
**5**	10^{-2}	0.20	50	$5.96 \cdot 10^{-4}$
**6**	10^{-1}	0.207	50	$6.17 \cdot 10^{-4}$
**7**	1	0.1	100	$5.96 \cdot 10^{-4}$

S3) Repeated extraction at different NaOH concentrations

The samples prepared using PEI-750k were repeatedly stained with pyranine and extracted in different concentrations of NaOH. Raw data presented here are from optical measurements.

Table S3-1. *Pyranine amounts for PEI-750k extracted with [NaOH]=0.01 M*

Cycle no.	E(455 nm)	v/ml	n/A / mole·m^{-2}
1	0.26	50	$7.75 \cdot 10^{-4}$
2	0.245	50	$7.3 \cdot 10^{-4}$
3	0.23	50	$6.85 \cdot 10^{-4}$
4	0.22	50	$6.56 \cdot 10^{-4}$
5	0.23	50	$6.85 \cdot 10^{-4}$
6	0.21	50	$6.85 \cdot 10^{-4}$
7	0.185	50	$5.51 \cdot 10^{-4}$
8	0.13	50	$3.88 \cdot 10^{-4}$
9	0.13	50	$3.88 \cdot 10^{-4}$
10	0.125	50	$3.73 \cdot 10^{-4}$

Table S3-2. *Pyranine amounts for PEI-750k extracted with [NaOH]=0.1 M*

Cycle no.	E(455 nm)	v/ml	n/A / mole·m^{-2}
1	0.225	50	$6.7 \cdot 10^{-4}$
2	0.245	50	$7.3 \cdot 10^{-4}$
3	0.235	50	$7.0 \cdot 10^{-4}$
4	0.1025	100	$6.13 \cdot 10^{-4}$
5	0.2	50	$5.96 \cdot 10^{-4}$
6	0.112	50	$3.34 \cdot 10^{-4}$
7	0.082	50	$2.44 \cdot 10^{-4}$
8	0.046	50	$1.37 \cdot 10^{-4}$
9	0.046	50	$1.37 \cdot 10^{-4}$
10	0.015	50	$0.45 \cdot 10^{-4}$

Table S3-3. *Pyranine amounts for PEI-750k extracted with [NaOH]=1 M*

Cycle no.	E(455 nm)	v/ml	n/A / mole·m^{-2}
1	0.115	100	$6.85 \cdot 10^{-4}$
2	0.08	100	$4.76 \cdot 10^{-4}$
3	0.004	100	$0.24 \cdot 10^{-4}$
4	0.003	100	$0.18 \cdot 10^{-4}$
5	0.004	100	$0.24 \cdot 10^{-4}$
6	0.002	100	$0.12 \cdot 10^{-4}$
7	0.003	100	$0.18 \cdot 10^{-4}$
8	0.003	100	$0.18 \cdot 10^{-4}$
9	0.001	100	$0.06 \cdot 10^{-4}$
10	0.002	100	$0.12 \cdot 10^{-4}$

S4) Variation of molar mass of PEI

The samples prepared using PEI of different molar mass were repeatedly stained with pyranine and extracted in 0.01 M NaOH. Raw data presented here are from optical measurements.

Table S4-1. *Pyranine amounts for M_w(PEI)=25 kg mol^{-1}*

Cycle no.	E*	v/ml	n/A /mol/m^{-2}	$\sigma_{relative}$/%
1	0.128	50	$3.82 \cdot 10^{-4}$	5.5
2	0.093	50	$2.76 \cdot 10^{-4}$	8.2
3	0.083	50	$2.48 \cdot 10^{-4}$	8.7
4	0.077	50	$2.3 \cdot 10^{-4}$	7
5	0.078	50	$2.33 \cdot 10^{-4}$	7.4

* Averaged from 4 values

Table S4-2. *Pyranine amounts for M_w(PEI)=2 kg mol^{-1}*

Cycle no.	E*	v/ml	n/A /mol/m^{-2}	$\sigma_{relative}$/%
1	0.074	50	$2.21 \cdot 10^{-4}$	6.3
2	0.078	50	$2.33 \cdot 10^{-4}$	14.2
3	0.076	50	$2.27 \cdot 10^{-4}$	5.1
4	0.046	50	$1.37 \cdot 10^{-4}$	5.2
5	0.049	50	$1.46 \cdot 10^{-4}$	5.6

* Averaged from 4 values

References

52 J.S. Lindsey, M. Taniguchi, H. Du, PhotochemCAD 1998-2023, https://photochemcad.com/databases/common-compounds/polycyclic-aromatic-hydrocarbons/pyranine, accessed 16 October 2023
